# Near-Fatal Multiple Organ Dysfunction Syndrome Induced by *Plasmodium malariae*

**DOI:** 10.3201/eid1505.081091

**Published:** 2009-05

**Authors:** Pierre-Néri Descheemaeker, Jean-Paul Mira, Fabrice Bruneel, Sandrine Houzé, Michèle Tanguy, Jean-Pierre Gangneux, Erwan Flecher, Christophe Rousseau, Jacques Le Bras, Yannick Mallédant

**Affiliations:** Hôpital Pontchaillou, Université de Rennes 1, Rennes, France (P.-N. Descheemaeker, M. Tanguy, J.-P. Gangneux, E. Flecher, Y. Mallédant); Hôpital Cochin, Port-Royal, Paris, France (J.-P. Mira, C. Rousseau); Centre Hospitalier de Versailles, Versailles, France (F. Bruneel); Hôpital Bichat Claude-Bernard, Paris (S. Houzé, J. Le Bras)

**Keywords:** Malaria, parasites, Plasmodium malariae, septic shock, ARDS, genetic polymorphism, letter

**To the Editor:** We report a case of *Plasmodium malariae*–related multiple organ dysfunction syndrome (MODS) in a healthy immunocompetent patient. Despite extensive investigation, *P. malariae* was the only pathogen identified. The patient’s isolates had a combination of mutant alleles that could possibly explain the severity of the infection.

Five weeks after returning to France in November 2006 from Côte d’Ivoire, a 28-year-old French soldier was admitted to our surgical intensive care unit (University Hospital, Rennes, France) because of fever and MODS of suspected infectious origin. The patient had stopped taking his doxycycline for antimalarial chemoprophylaxis 3 days before his admission. During those 3 days he began to experience myalgia, fatigue, nausea, and vomiting but no fever. He took no medication. He then became unable to move his lower legs and experienced paresthesia just before his condition rapidly deteriorated. He was found at home by the local Emergency Medical Service (EMS) in respiratory distress and shock and required immediate orotracheal intubation for mechanical ventilation. When admitted to the intensive care unit, he had severe acute respiratory distress syndrome (PO_2_/FiO_2_, 65 mm Hg; PCO_2_, 90 mm Hg; with positive end expiratory pressure of 12 cm H_2_O). Transthoracic echocardiography and pulmonary artery catheterization showed severe global hypokinesia with a left ventricular ejection fraction of <10%, right ventricular dilatation, and low pulmonary artery occlusion pressure. Blood tests showed disseminated intravascular coagulation with 30 × 10^9^/L platelets, an international normalized ratio of 3.54, an activated partial thromboplastin time >180 s, and D-dimers at 25.6 µg/mL. He had severe mixed acidosis (pH 6.9 and arterial lactate 4.2 mmol/L) and acute renal failure. Blood cultures were performed. A thin-blood film showed *Plasmodium* spp. within the red blood cells (parasitemia 0.4%). Rapid fluid resuscitation was carried out and epinephrine was given, along with intravenous quinine (1,000 mg over 4 h, then 1,500 mg/d) and broad-spectrum antimicrobial drugs (cefotaxime and ofloxacine).

Massive acidosis developed (pH 6.61; lactate 8.8 mmol/L). A brief cardiac arrest required chest compressions and extracorporeal membrane oxygenation (ECMO) after venoarterial femoral cannulation at the bedside. Continuous venovenous hemofiltration was started. APACHE II and SAPS 2 scores were 38 and 93, respectively. Drotrecogin-alpha (activated) was given as a 96-h infusion.

Extensive microbiologic investigations included tests for common bacteria at usual sampling sites and tests for specific arboviruses, *Leptospira* spp., *Rickettsia* spp., and parasites other than *Plasmodium* spp. Results were negative. *P. malariae* was found in the thick-blood film.

The patient’s cardiac and pulmonary functions stabilized over the next week. Epinephrine and ECMO were stopped. Surgical exploration of extensive lower-limb necrosis showed arterial thrombosis and *Serratia marcescens* infection. Amputation was necessary of the right leg at the thigh and of the left lower leg after 1 month of hospitalization. The patient was discharged from intensive care 90 days after hospital admission. One year later he was fully recovered and was using prostheses.

Almost all deaths from malaria are related to *P. falciparum* infection. We are unaware of previous reports of near-fatal imported *P. malariae* infection (*1*). Although we cannot definitively exclude bacterial co-infection, results were negative from blood cultures drawn before the first antimicrobial drug dose and from all other microbiologic tests. To look for factors that might explain the unusual disease severity, we conducted additional investigations. The thick blood film showed *P. malariae* trophozoites, schizonts, and gametocytes (285/µL). Rapid diagnostic tests were positive for pan–*Plasmodium* lactate dehydrogenase (pLDH) and aldolase but negative for histidine-rich protein 2, which is specific for *P. falciparum*. Nested PCR with specific primers for *P. falciparum* and *P. malariae*, followed by sequence analysis of the SSUrRNA gene, and nested PCR, followed by sequence analysis of the pLDH gene, were negative for *P. falciparum*, positive for *P.* *malariae*, and negative for *P. knowlesi* (*2*).

The patient gave written informed consent to tests for genetic polymorphisms associated with severe sepsis, coagulation disorders, and MODS (*3*). Several of these polymorphisms were found (Table). He had a mannose-binding lectin (MBL) haplotype associated with low MBL levels (2 variants in the promoter region [homozygous at –550 and heterozygous at –221] and 1 in the first exon [heterozygous for codon 54 mutation]) (*5*). He was homozygous for 4 gene polymorphisms associated with higher susceptibility or severity of severe sepsis or both and ARDS: *CD14***,** lymphotoxin alpha, *TNF-alpha, IRAK-1,* and *IL-6* (*6**–**9*). Furthermore, he was homozygous for a *PAI-1* variant associated with decreased fibrinolysis and a higher risk for amputation, skin grafting, and death in meningococcal disease and trauma (*10*). Of 11 patients with uncomplicated *P. malariae* whom we screened for these polymorphisms, none had such a combination of high-risk polymorphisms as did our patient. Thus, the genetic background of our patient may have contributed to the severity of *P. malariae* infection.

**Table:** Results of genetic screening for single nucleotide polymorphisms known to be associated with sepsis severity in patient with *Plasmodium malariae* infection, France*

Gene	rs	Wild type	Heterozygous	Homozygous
Pathogen detection				
TLR2	5743708	X		
TLR2	5743704	X		
TLR4	4986790	X		
TLR5	5744168	X		
CD14	2569190			X
MD-2	–1625C/G	X		
FcγRIIa	1801274		X	
MBL2	52 A/D	X		
MBL2	54 A/B		X	
MBL2	57 A/C	X		
MBL2	–550 H/L			X
MBL2	–221 X/Y		X	
MBL2	+4 P/Q	X		
TLR signaling				
IRAK1	1059703			X
TIRAP	8177374		X	
IκB	3138053		X	
IκB	2233406		X	
Inflammation				
Lymphotoxin α	909253			X
TNF α	1800629			X
ACE	17236674			X
MIF	755622		X	
IL-6	1800795			X
IL-10	1800896	X		
Coagulation				
PAI-1	1799768			X
Factor V	6025	X		

*Except for MD-2 and MBL2, rs is the nomenclature used to describe the variants (initially described by den Dunnen and Antonarakis [*4*]). TLR, toll-like receptor; MBL2, mannose-binding lectin 2; IRAK-1, interleulin-1 receptor-associated kinase; TIRAP, toll interleukin-1 receptor-associated protein; IκB:, inhibitor of Nf-κB; TNF, tumor necrosis factor; ACE, angiotensin-converting enzyme; MIF, macrophage migration inhibitory factor; IL, interleukin; PAI-1, plaminogen activator inhibitor-1.

Despite extensive testing, we found no cause for this near-fatal case of MODS except *P. malariae* infection. An unusual combination of genetic polymorphisms may explain the extreme severity of this classically mild infection.
